# Nutritional and Culinary Habits to Empower Families (n-CHEF): a feasibility study to increase consumption and home cooking of plant-based foods

**DOI:** 10.1017/S1368980024001538

**Published:** 2024-10-01

**Authors:** Leticia Goni, Luca Simonin, Anacristina Rovayo, Isabella Kury-Guzman, Nerea Martín-Calvo, Miguel Ruiz-Canela

**Affiliations:** 1 Department of Preventive Medicine and Public Health, IdiSNA (Instituto de Investigación Sanitaria de Navarra), University of Navarra, Pamplona, Spain; 2 CIBER Fisiopatología de la Obesidad y Nutrición (CIBERObn), Instituto de Salud Carlos III, Madrid, Spain

**Keywords:** Culinary nutritional intervention, Parent–child dyad, Plant-based foods, Mediterranean diet, Feasibility study

## Abstract

**Objective:**

To analyse the feasibility and acceptability of a culinary nutritional intervention aimed at increasing plant-based foods consumption in the context of the Mediterranean diet in parent–child dyads.

**Design:**

The Nutritional and Culinary Habits to Empower Families (n-CHEF) is a 9-month feasibility study that included four culinary nutritional workshops (two face to face, two online) led by a chef and a dietitian-nutritionist. These workshops combined cooking with plant-based foods, with nutritional advice and experimental activities. The main outcomes were retention, quality of the intervention (monitoring workshops, acceptability and perceived impact) and changes in dietary and cooking habits.

**Setting:**

Parent–child dyads, Spain.

**Participants:**

Parent–child (aged 10–14 years) dyads.

**Results:**

Fifteen parent–child dyads were recruited, of which thirteen were retained during the 6-month follow-up. All but one parent–child dyads attended the four workshops. The overall assessment of the workshops was positive, although the online workshops were rated lower than the face to face. In general, parent–child dyads reported benefits in terms of nutrition and cooking aspects. Parents significantly increased their adherence to the Mediterranean diet, but non-significant changes were observed in children. However, children increased their consumption of vegetables and legumes and reduced snacks and ready meals. Parents also changed some of their culinary habits and increased their confidence in cooking at home.

**Conclusions:**

The n-CHEF showed that the culinary nutritional intervention had good levels of recruitment, retention and acceptability among parent–child dyads. In addition, dietary and culinary knowledge and habits can be improved, although further studies are needed to know the long-term effects in larger populations.

The population has more and more information about the importance of eating habits for a healthy life^(1)^. However, the current high prevalence of overweight and obesity in childhood^(2)^ and other chronic conditions such as metabolic syndrome^(3)^ suggests that, among other factors, there is a gap between the information available and the dietary habits, especially the consumption of plant-based foods^(4,5)^. One of the factors that has been identified as a barrier to maintaining a healthy diet is limited knowledge, skills and abilities related to home cooking^(6)^. In addition, the home environment has a strong influence on the development and maintenance of children’s eating habits^(7)^. Indeed, parental attitudes, beliefs, behaviours and decisions can influence children’s dietary choices^(8,9)^, as well as the development of childhood overweight^(10,11)^.

Family-focused public health is based on the idea that health begins at home, and the family environment has a relevant role in the healthy development of children at primary, secondary and tertiary prevention levels^(12)^. In fact, previous studies have demonstrated that family-based nutrition interventions can improve health and dietary habits^(13)^. Dietary interventions aiming to improve the consumption of plant-based foods among children have reported a positive effect on increasing vegetables and fruits^(14,15)^ while others did not^(16)^. In this context, promoting home cooking can be an effective strategy for chronic disease prevention and health promotion^(17–19)^ by improving children’s dietary habits^(20–23)^. However, most of these studies have been conducted in the USA^(20–22,24–26)^, with short follow-up (<3 months)^(20,22,24,26–28)^ and a limited information about the dietary changes among parents and children^(26–29)^. In person and online cooking courses (both free or subscription-based) are available for families^(30,31)^, although to the best of our knowledge there is no information on the effectiveness of these courses. Online resources may also be beneficial for promoting home cooking, but further studies are needed to evaluate the long-term utility of these tools with families^(32)^.

Home cooking interventions could offer additional benefits to families with children beyond interventions that provide only nutritional recommendations. This assumption is based on the fact that it reinforces their ability to make nutritional changes through healthy home-cooked meals, reinforces the knowledge they have acquired and could allow for more lasting behavioural changes. However, previous studies have not provided adequate information about the culinary interventions and/or the professionals involved^(22,24,27,28)^. In addition, the provision of theoretical background that may support the behavioural change of the participants is missing^(33)^. All this information is needed to assess the feasibility of these interventions which usually are more complex in terms of the resources needed and the time and availability of the participants.

In this context, we present the results of the Nutritional and Culinary Habits to Empower Families (n-CHEF) feasibility study. In this study, we evaluated the effect of a hands-on culinary and nutritional intervention involving at least one parent and one child in a kitchen with a dietitian-nutritionist and a chef after over a 9-month period. The main objective was to assess the feasibility, acceptability and efficacy of the culinary and nutritional intervention aimed at increasing the consumption of plant-based foods in the context of the Mediterranean diet and the use of healthy cooking techniques. In addition, we provide a detailed description of the culinary-nutritional intervention conducted in the n-CHEF feasibility study.

## Methods

### Study design and participants

The n-CHEF feasibility study was carried out at the Department of Preventive Medicine and Public Health of the University of Navarra. The total follow-up was 9 months, and it was divided into two periods: the intervention period (April–June 2021, 3 months) during which parent–child dyads participated in four culinary nutritional workshops (two face-to-face and two online) and the follow-up period (July–December 2021, 6 months).

A total of fifteen dyads of a parent and a child were recruited through an advertisement published in March 2021 in the weekly online newsletter for the employees of the University of Navarra. As the n-CHEF is a feasibility study, sample size calculation was not applied, but we estimated that fifteen parent–child dyads would allow us to find some significant intra-subject differences between baseline and follow-up^(34)^. The main inclusion criteria were families with an available progenitor and a healthy child aged 10–14 years, living in the same household. The narrow age range for children was chosen in order to design a culinary and nutritional intervention that was as age appropriate as possible in terms of theoretical and practical content. Exclusion criteria were having attended previous cooking courses, food allergies or intolerances incompatible with the culinary nutritional intervention, following a specific dietary pattern, or having a chronic disease including eating disorders.

The trial was registered on ClinicalTrials.gov NCT04986449.

### Culinary nutritional intervention

**Fig. 1:**
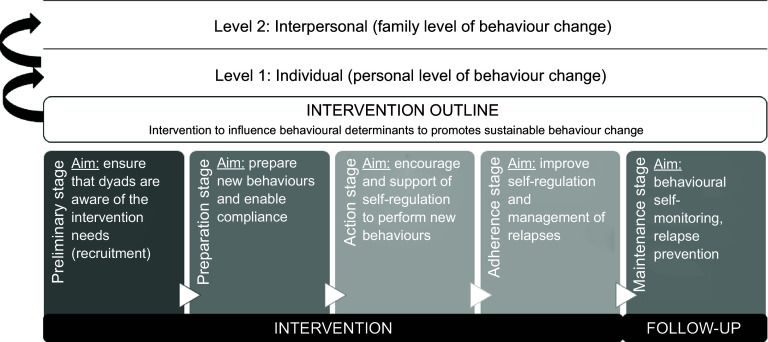
Logic model of behaviour change of the n-CHEF intervention. N-CHEF, Nutritional and Culinary Habits to Empower Families

The primary aim of the culinary and nutritional intervention was to promote the consumption of plant-based foods in the context of the Mediterranean diet. The Mediterranean diet is characterised by the exclusive consumption of extra virgin olive oil for all culinary purposes and high consumption of vegetables, fruits, legumes, nuts and whole grains; moderate consumption of fish and very low consumption of red and processed meat, refined grains, sweet desserts, whole-fat dairy products (only consumed in moderation fermented dairy products such as yogurt and cheese) and ultraprocessed foods. The intervention was designed by a multidisciplinary group, including a chef, dietitian-nutritionists and an epidemiologist following the ten experiential drivers of behaviour change in culinary nutrition education identified by Fredericks and cols.^(35)^. The ten experiential drivers are challenge (trying new foods/flavors and skills), celebration (creating fun, enjoyable and special atmosphere; creating deliciousness from healthy food), collaboration (generating positive group dynamic, a feeling of being part of something bigger), home environment (addressing home dynamics, facilities and access to healthy food; creating solutions and strategies), palate development (tasting a wide range of flavors, create new combinations), peer support (creating an atmosphere of playing level field where new behaviour becomes acceptable), recipe concept (recipe-driven cooking, recipe concepts and ingredients swap), skill building (developing new culinary skills and vocabulary, motivating to share new food strategies with others), skill reinforcement (measuring participants advancing skills) and success (achieving goals, increase participant confidence, competency and sense of accomplishment). The content and activities proposed in the workshops followed at least one of the experiential drivers identified by Fredericks and cols.^(35)^ to motivate participants towards positive changes in food behaviours. Table 1 lists the experiential drivers applied in each workshop with an example of the content or activity. Figure 1 shows the proposed logic model explaining how behaviour change is expected to occur as a result of the n-CHEF intervention.

**Table 1:** Workshops’ description of the n-CHEF intervention

**Workshop 1** (Face-to-face)	*Recipes*	Pumpkin and orange cream/Leek steamed, carrot cream and roasted peanuts/Parsnip chips and sweet potato “fries”, and *Romescu* sauce/Vegetable broth/Sautéed seasonal mushrooms and mashed potato/Marinated strawberries with healthy cocoa cream
	*Food groups present*	Fruits, vegetables, nuts, oils
	*Culinary techniques used*	Types of cutting, baking, sautéing, boiling, toasting, marinade, *Papillote*
	*Experiments and activities*	Why does food taste better when we cook it? (*Maillard* reaction experiment), ‘Mushrooms in the Sun’, potato glue, why do certain fruits oxidize?, vegetable butter, oils tasting, video about the production of extra virgin olive oil, water with sugar or coke ¿which is sweeter?
	*Cooking concepts*	*Mise en place*, preparation of broths, *Maillard* reaction, cooking starchy foods, full utilisation of vegetables, marinade (food preservation without affecting taste), flavour enhancers, fat smoke point, flavor/textures according to vegetable maturation, use sugars from foods – healthier alternatives to sugar in recipes
	*Nutritional concepts*	Why we cook foods, bioavailability and loss of nutrients according to the culinary technique used, types of fats, types and properties of olive oils, vitamins D, E and A, and antioxidants, nutritional and health properties of nuts, types of sugar and alternatives to sugar, health properties of fibre, properties of vegetables according to their maturation
	*Experiential drivers of behaviour change*	Challenge (e.g. oils testing), celebration (e.g. healthy cocoa cream), collaboration (e.g. cooking as a family), home environment (e.g. full utilisation of vegetables), palate development (e.g. marinade), peer support (e.g. all participants cutting), recipe concept (e.g. healthier alternatives to sugar in recipes), skill building (e.g. *Maillard* reaction), success (e.g. cutting foods)
**Workshop 2** (Face-to-face)	*Recipes*	Peas with yogurt and mint/Zucchini noodles with pistachio pesto/White asparagus with poached onion, *Ajoblanco* (Spanish source based on garlic, almonds and extra virgin olive oil)/*Pisto* (Spanish vegetable dish of chopped tomatoes, pepper, eggplant, onion among others stewed together) with egg/Baked apple with cinnamon and healthy meringue
	*Food groups present*	Fruits, vegetables, oils, nuts, dairy products, eggs, herbs and spices
	*Culinary techniques used*	Types of cutting, baking, roasting, sautéing, boiling, *Papillote,* microwaving, infusing, blanc
	*Experiments and activities*	How to ripen food based plants, blind tasting: colour and taste, onion, a tear gas, cinnamon experiment (flavour), blind tasting: aroma *v*. essences, what is an emulsion? How do I know if the egg is bad? Foodpairing web page
	*Cooking concepts*	Tips to combine tastes or foods according to their colours, types of spiciness, cook with herbs and spices, culinary techniques to cook egg, elaboration process of cheese and butter, how to include cheese in recipes, blanc for vegetables and fruits, types of emulsions, emulsions in recipes, healthier options to cream in recipes
	*Nutritional concepts*	Seasonal and local products, food colours and aromatic compounds, taste, smell and flavour, appetite, differences between processed and ultra-processed foods, nutritional and health properties of dairy products and egg, probiotics and prebiotics, fat sources (mayonnaise), ratio omega 3:omega 6
	*Experiential drivers of behaviour change*	Challenge (e.g. blind tasting), celebration (e.g. food colours), collaboration (e.g. cooking as a family), home environment (e.g. food pairing), palate development (e.g. cinnamon experiment), peer support (e.g. all participants cleaning), recipe concept (e.g. seasonal and local products), skill building (e.g. flavour), skill reinforcement (e.g. types of cutting), success (e.g. cooking with microwave)
**Workshop 3** (Online)	*Recipes*	Fast bread/Whole wheat and spelt loaf/Whole grain noodles with pea sauce and snow peas/Dried tomato and mozzarella ravioli with almond and sage sauce/Unsweetened banana and cocoa marble cake
	*Food groups present*	Fruits, vegetables, oils, nuts, dairy products, egg, whole grain cereals
	*Culinary techniques used*	Bread elaboration, pasta elaboration, sautéing, voiling, baking
	*Experiments and activities*	Why does my cake/bread rise?, *Kahoot!* (quizzes)^(36)^
	*Cooking concepts*	Sourdough, Fermentation process of breath and other doughs, types of yeast, bread and pasta elaboration
	*Nutritional concepts*	Nutritional and health benefits of whole grain cereals *v*. refined cereals, nutritional and health benefits of unsweet desserts
	*Experiential drivers of behaviour change*	Challenge (e.g. sourdough), celebration (e.g. ravioli), collaboration (e.g. in group workshop), home environment (e.g. whole grain cereals), palate development (e.g. unsweetened cake), peer support (e.g. all participants kneading bread), recipe concept (e.g. types of yeast), skill building (e.g. fermentation), skill reinforcement (e.g. sautéing) success (e.g. *Kahoot!*)
**Workshop 4** (online)	*Recipes*	Peach *Salmorejo* (Spanish cream usually based on tomato, extra virgin olive oil, bread and garlic)/Chickpea and almond hummus / Beetroot, apple and avocado tartare/Energy balls for snack (carrot, dates and coconut)
	*Food groups present*	Fruits, vegetables (canned or frozen), legumes (canned), oils, nuts
	*Culinary techniques used*	Grinding
	*Experiments and activities*	Focus group barriers and resources cooking at home, prepare your weekly healthy menu, *Kahoot!* (quizzes)^(36)^
	*Cooking concepts*	Quick and healthy recipes, used of 5^th^ range foods, recommendations of recipe books, social media accounts related to cook
	*Nutritional concepts*	Batch cooking, weekly healthy menu planning, recommendations education nutrition books, social media accounts related to nutrition, recommendations on nutritional labelling and food purchase
	*Experiential drivers of behaviour change*	Challenge (e.g. quick recipes), celebration (e.g. energy balls for snack), collaboration (e.g. in group workshop), home environment (e.g. food purchase), palate development (e.g. raw foods), peer support (e.g. all participants suggesting barriers, resources), recipe concept (e.g. batch cooking), skill building (e.g. menu planning), skill reinforcement (e.g. healthy recipes) success (e.g. *Kahoot!*)

n-CHEF, Nutritional and Culinary Habits to Empower Families.

**Fig. 2:**
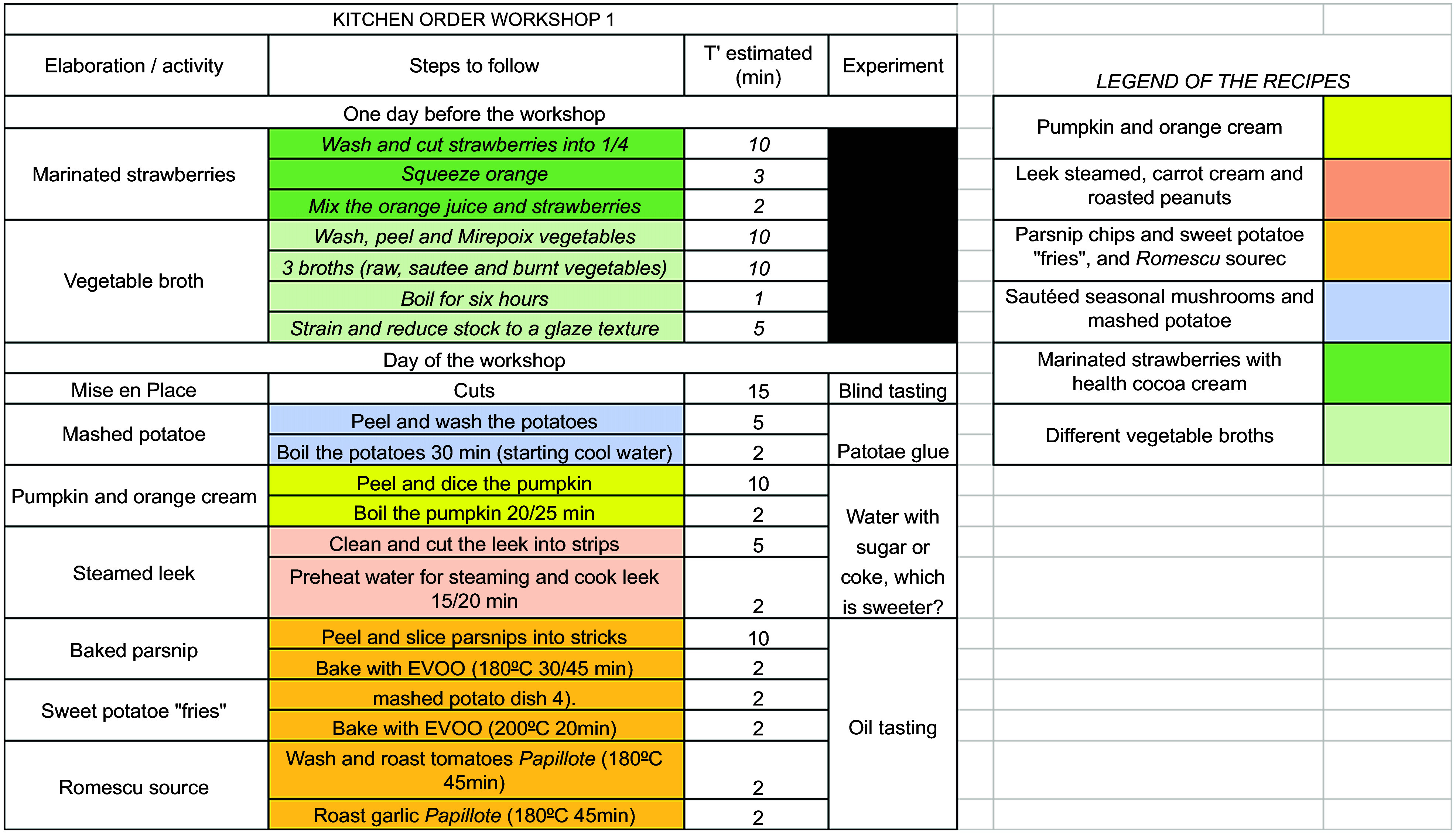
Screenshot of the Excel sheet prepared by the researchers with the content and the timeline of each workshop

During the intervention period, each parent–child dyad attended four workshops and the final n-CHEF meeting (Table 1). The dyads attended the workshops every 4 weeks. Workshops 1 and 2 were conducted face to face for each dyad, and the duration of each was set at 3 h. Workshops 3 and 4 were held online via Zoom in groups of five parent–child dyads and the duration was set at 2 h. We decided to include both face-to-face and online workshops in order to assess the feasibility of both types of intervention. The research team provided multiple option dates to follow-up the workshops to overcome barriers related to unreliable work schedules and children’s schedules. For the final n-CHEF meeting, parent–child dyads were gathered face-to-face on the same day. A chef and a dietitian-nutritionist conducted all the workshops and the final meeting. The research team designed an Excel sheet with the content and the timeline of each workshop (Fig. 2). Before the first workshop, a training session was organised with a dyad (parent–children) not included in the study. In this training session, the chef and the dietitian-nutritionist were able to implement the training plan (nutritional and cooking concepts, recipes, experiments and other activities) devised for the workshop.

All workshops were conducted in a kitchen, and they included culinary and nutritional information, both from a theoretical and practical perspective. Parent–child dyads cooked different meals and participated in different activities described in Table 1. In the first workshop, the main objective was to show the nutritional and health properties of plant-based foods in general, and vegetables in particular. After the families experimented with the smells, tastes and colours of different vegetables, they cooked them. The aim of the second workshop was to introduce the families to the concept of sustainable diet where seasonal and local foods should be the foods of choice^(37)^. In this workshop, the families cooked vegetables with other less common culinary techniques and learned how to use different spices and aromatic herbs to make their dishes different, avoiding the idea that choosing seasonal and local foods is monotonous. In the third workshop, the aim was to replace the use of refined grains with whole grains^(38)^, taking into account that whole grains are less accepted by children, and that they are differently cooked than refined grains. Finally, the fourth workshop aimed to introduce the concept of weekly menu planning including the shopping list, the idea of batch cooking and the use of kitchen pantry foods (canned legumes, nuts, seeds) and other frozen home preparations (*sofrito*, fish broth).

Regarding the economic cost of the culinary nutritional intervention, each family paid for the shopping list provided to them before each workshop except for basic ingredients (salt, whole flour, extra-virgin olive oil and aromatic herbs and spices) and cooking utensils which were provided by the researchers.

### Clinical visits

During the study parent–child dyads attended three clinical visits: at baseline (0 months), at the end of the intervention period (3 months) and at the end of the follow-up period (9 months in total) in the facilities of the University of Navarra.

#### Baseline measurements

At the baseline visit, sociodemographic information and family and medical history were collected from the children. Anthropometric measurements were obtained from both the progenitor and the child, at baseline and at the end of the follow-up period, in light clothing and without shoes. Body weight was measured using the Tanita RD-545 (Tanita Corp), and height was measured to the nearest 1 mm using a portable stadiometer. BMI was calculated as body weight (kg)/height (m)^2^. For children, the BMI-for-age (BMI/A) *z*-score was also calculated according to the 2007 WHO growth charts (38). Waist and hip circumference were measured with a flexible tape at the midpoint between the last rib and the edge of the iliac crest and around the maximum circumference of the buttocks, respectively. Finally, children were asked about their physical activity using a previously validated questionnaire for adults^(39)^.

#### Dietary measurements

The children’s dietary habits were analysed at baseline and at the end of the follow-up period using a 147-item semi quantitative FFQ validated for the Spanish pediatric population^(40)^. Energy and nutrient intakes were derived from Spanish food composition tables^(41–43)^.

Adherence to the Mediterranean diet was assessed from children and adults using the validated seventeen-item Mediterranean diet adherence score^(44)^. For children, this score was derived from the FFQ but without the question on wine consumption. Mediterranean diet adherence was measured in children, at baseline and after the follow-up period, whereas in adults, it was measured at baseline, after the intervention period and after the follow-up period.

#### Culinary measurements

Confidence and attitudes about home cooking from progenitors were evaluated at the three in person visits using a self-administered questionnaire adapted from Condrasky and cols.^(45)^ and Vrhnovnik^(46)^. In addition, the Home Cooking Frequency Questionnaire previously validated in adults was administered to progenitors at baseline and at the end of the study^(47)^.

### Feasibility evaluation

#### Inclusion and retention

We evaluated the recruitment process to identify the potential barriers to family engagement in the project. We also measured the retention rate ((number of participants who completed the trial/total number of participants) × 100) and recorded the reasons for dropout and attrition to identify potential barriers to retention during the 3-month intervention period and the additional 6-month follow-up.

#### Quality evaluation of the intervention

The research team monitored parent–child dyad attendance, timing and economic costs of each workshop. In addition, after each workshop, a satisfaction questionnaire, developed *ad hoc* for this study, was sent to parent–child dyads to assess the acceptability of the intervention in terms of quality, durability, content and development of each workshop. This questionnaire included questions using a five-point Likert scale, such as ‘The duration is adequate’, ‘We enjoyed it’, ‘The methodology used has facilitated the active participation of all the family’…; and open-ended questions such as ‘What did you like the best/least?’ and ‘What would you change?’.

In addition, at the end of the intervention, both parents and children self-administered an *ad hoc* questionnaire aimed to measure the changes in perceptions and attitudes in relation to home cooking. This questionnaire, using a three-point Likert scale, included questions for children such as ‘Now I like vegetables, fruits, whole grains, legumes more than when I started the workshops’, ‘After the study, I help my parents, more cook than before’…; and for parents such as ‘The n-CHEF study helped me to increase the amount of vegetables we cook at home’, ‘The n-CHEF study helped me to increase my awareness of the need to buy local, seasonal products, and to limit waste’…

#### Outcomes of the intervention

In the feasibility study, we aimed to evaluate changes in dietary and culinary habits in both parents and children. The primary outcome was to measure changes in the adherence to the Mediterranean diet after the intervention in children and parents (seventeen-item Mediterranean diet adherence score^(44)^). The secondary outcomes were perceived benefits of the intervention by children and parents, changes in dietary habits in children after the total 9-month follow-up and changes in culinary habits and cooking confidence and attitudes in parents after the 3 month-intervention period and the additional 6-month maintenance period.

### Statistical analysis

Categorical variables were described as absolute numbers and percentages and quantitative variables as mean and sd. The Shapiro–Wilk test was used to test the normal distribution of the quantitative variables. Within-subject changes in dietary and culinary habits were analysed using the Student’s *t*-test for paired data or the Wilcoxon matched-pairs signed-rank test, depending on the distribution of each variable. Changes in quantitative variables were as mean and 95 % CI. Statistical analyses were performed with STATA software (STATA version 16·0, StataCorp). All *P* values <0·05 were considered statistically significant.

## Results

### Enrollment and retention

**Fig. 3:**
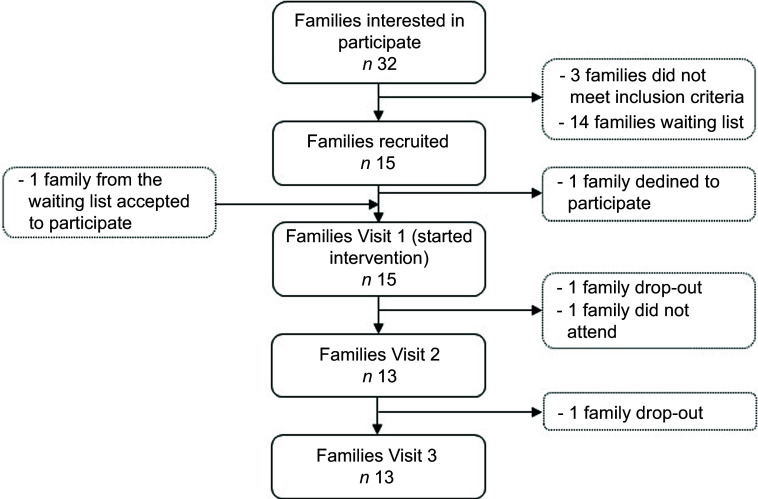
Flow chart of the n-CHEF feasibility study participants (parent–child dyads). N-CHEF, Nutritional and Culinary Habits to Empower Families

A total of thirty-two parent–child dyads were interested in participating on the same day that information about the study was disseminated at the University of Navarra (Fig. 3). The first fifteen parent-child dyads that met the inclusion criteria were included in the feasibility study. Three parent–child dyads did not meet the inclusion criteria and the remaining fourteen parent–child dyads were placed on a waiting list. One parent–child dyad refused to participate because the intervention did not fit into with their daily activities prior to the start of the intervention. Therefore, one parent–child dyad from the waiting list was invited to participate in the study. Finally, a total of fifteen parent–child dyads started the intervention. During the study two parent–child dyad were drop-out (1 at clinical visit 2 and 1 at clinical visit 3). Thus, results during the follow-up (including the 3-month intervention period and the additional 6-months) were available only for thirteen parent–child dyads, being the retention rate 86·7 %.

### Baseline characteristics

Baseline characteristics of the parent–child dyads participating in the feasibility study are shown in Table 2. Eighty percent of the children and 53·3 % of the adults were female. The mean (sd) age was 12·6 (1·3) and 46·5 (5·2) years for children and parents, respectively. A high proportion of the parents in the study had college education (93·3 %), and 66·7 % of the families had three or more children. The mean BMI/A (sd) for children was 0·5 (0·7) *z*-score, and the BMI mean (sd) for parents was 24·9 (3·7) kg/m^2^.

**Table 2:** Baseline characteristics of the parent–child dyads recruited in the n-CHEF feasibility study

	Children (*n* 15)		Adults (*n* 15)	
	Mean	sd	Mean	sd
Sex, female*	12	80·0	8	53·3
Age (years)	12·6	1·3	46·5	5·2
Education level, university studies*	N/A		14	93·3
Eat at cafeteria*	4	26·7	N/A	
Number of children ≥3*	N/A		10	66·7
Weight (kg)	48·0	6·9	71·2	14·7
Height (m)	1·57	9·7	1·69	8·5
BMI	19·5	1·8	24·9	3·7
BMI/A	0·5	0·7	–	
Waist circumference (cm)	69·0	5·1	88·3	12·6
Hip circumference (cm)	86·3	6·2	103·0	8·5
Physical activity (MET-hours/week)	34·1	24·0	N/A	

BMI/A, BMI-for-age; MET, metabolic equivalents; NA, not applicable; n-CHEF, Nutritional and Culinary Habits to Empower Families.

* Data are *n* (%).

### Acceptability of the intervention

The fifteen parent–child dyads attended all of the workshops except for one parent–child dyad who did not attend the fourth workshop and two parent–child dyads who did not attend the final meeting. Initially, the duration of each workshop was set at 3 h for the face-to-face workshops (1 and 2) and 2 h for the online workshops (3 and 4). Workshops 1, 2 and 4 lasted the specified time. However, workshop 3 lasted 45 min longer than the established time of two hours. The average cost of the shopping list per workshop was 17€ (five recipes for four people each), whereas the total cost of the materials used in the intervention for the research team was 823€.

Data from the *ad hoc* satisfaction questionnaire after each workshop were analysed to determine the acceptability of the intervention by the parent–child dyads (Table 3). In general, all workshops were rated highly by most of the dyads in terms of meeting expectations, level of depth of the topics covered, duration, methodology and usefulness of workshops content for everyday life. The workshop that was rated the lowest was the third workshop (on line) in terms of duration, methodology and usefulness of the content. Most parent–child dyads rated workshops 1, 2 and 4 as very good or excellent. However, workshop 3 was rated as good by 44·4 % of the parent–child dyads.

**Table 3:** Acceptance of the culinary and nutritional intervention by families

	Totally disagree/ disagree		Neutral		Agree		Totally agree	
	*n*	%	*n*	%	*n*	%	*n*	%
The content of the workshop has met our expectations								
Workshop 1*	0	0·0	0	0·0	2	15·4	11	84·6
Workshop 2*	0	0·0	0	0·0	4	30·8	9	69·2
Workshop 3†	0	0·0	3	33·3	4	44·4	2	22·2
Workshop 4‡	0	0·0	1	16·7	3	50·0	2	33·3
The level of depth of the topics covered has been adequate								
Workshop 1*	0	0·0	0	0·0	2	15·4	11	84·6
Workshop 2*	0	0·0	0	0·0	0	0·0	13	100
Workshop 3†	0	0·0	2	22·2	5	55·6	2	22·2
Workshop 4‡	0	0·0	2	33·3	2	33·3	2	33·3
The duration of the workshop has been adequate								
Workshop 1*	1	7·7	1	7·7	2	15·4	9	69·2
Workshop 2*	0	0·0	0	0·0	0	0·0	13	100
Workshop 3†	2	22·2	2	22·2	3	33·3	2	22·2
Workshop 4‡	0	0·0	1	16·7	3	50·0	2	33·3
The methodology used has allowed an active participation								
Workshop 1*	0	0·0	0	0·0	1	7·7	12	92·3
Workshop 2*	0	0·0	0	0·0	0	0·0	13	100
Workshop 3†	2	22·2	3	33·3	2	22·2	2	22·2
Workshop 4‡	0	0·0	3	50·0	1	16·7	2	33·3
The teachings we received are useful for our daily lives								
Workshop 1*	0	0·0	1	7·7	2	15·4	10	76·7
Workshop 2*	0	0·0	0	0·0	0	0·0	13	100
Workshop 3†	3	33·3	0	0·0	6	66·7	0	0·0
Workshop 4‡	0	0·0	1	16·7	3	50·0	2	33·3
The quality of the audio and video has been adequate (only online workshops)								
Workshop 3†	1	11·1	4	44·4	2	22·2	2	22·2
Workshop 4‡	1	16·7	2	33·3	1	16·7	2	33·3

	Bad/ Regular		Good		Very good		Excellent	
	n	%	n	%	n	%	n	%
The workshop deserves a ranking of …								
Workshop 1*	0	0·0	0	0·0	1	7·7	12	92·3
Workshop 2*	0	0·0	0	0·0	4	30·8	9	69·2
Workshop 3†	0	0·0	4	44·4	4	44·4	1	11·1
Workshop 4‡	0	0·0	1	16·7	2	33·3	3	50·0

* Thirteen responded of fifteen attended.

† Nine responded of fifteen attended.

‡ Six responded of fourteen attended.

### Perceived impact of the intervention

The results of the perceived benefits of the intervention by children and parents are shown in Table 4. After the intervention, most of the children agreed that they had improved their knowledge about cooking (84·6 %) and nutrition (92·3 %). However, 61·5 % of children perceived that their taste for plant foods (vegetables, fruits, legumes and nuts) had not improved as a result of the workshops. In addition, 69·2 % of the children agreed that they do not help their parents cook more often after the intervention. As for the parents, more than 80 % agreed that the intervention helped them to increase the variety of foods (84·6 %), involve their children more in cooking at home (92·3 %), become more aware of nutrition’s impact on health status (92·3 %), become more interested in cooking and gastronomy (92·3 %) and be more conscious on buying local and seasonal products and reducing food waste (84·6 %). However, a substantial proportion of parents were neutral or disagreed that the intervention helped them to cook more at home (38·4 %), reduce the consumption of convenience foods (23·0 %), increase the variety of vegetables cooked at home (30·8 %) and increase the consumption of whole grains (30·8 %).

**Table 4:** Perceived impact of the intervention on the children’s and parents’ attitudes towards cooking and nutrition*

Children. *After the workshop…*	Disagree		Neutral		Agree	
	*n*	%	*n*	%	*n*	%
I have more knowledge about cooking than before	1	7·7	1	7·7	11	84·6
I have more knowledge about nutrition than before	1	7·7	0	0·0	12	92·3
I like plant-based foods more than before	1	7·7	7	53·8	5	38·5
I help my parents on cooking at home more than before	1	7·7	8	61·5	4	30·8

Adults. *The n-CHEF study has helped to me to…*	Disagree		Neutral		Agree	
	*n*	%	*n*	%	*n*	%
Increase home cooking	1	7·7	4	30·7	8	61·6
Decrease the consumption of ready to eat foods	1	7·7	2	15·3	10	77·0
Increase the variety of foods cooked at home	0	0·0	2	15·4	11	84·6
Increase the quantity of vegetables cooked at home	1	7·7	3	23·1	9	69·2
Increase the quantity of whole grain cereals cooked at home	1	7·7	3	23·1	9	69·2
Involve children more in home cooking	0	0·0	1	7·7	12	92·3
Be more aware of the importance of nutrition on my health and that of my family	0	0·0	1	7·7	12	92·3
Have more interest on cooking and gastronomy	0	0·0	1	7·7	12	92·3
Be more aware on the use of local and seasonal products and food waste	0	0·0	2	15·4	11	84·6

* Data from the thirteen families that participated in the four workshops during the 3-month intervention period.

### Adherence to the mediterranean diet and changes in dietary and culinary habits

At baseline, the adherence to the Mediterranean dietary pattern, mean (sd), was 5·2 (2·2) for children and 8·1 (1·6) for parents. The Mediterranean diet adherence did no significant changed in children during the total 9-month follow-up (increase, 0·6 (–0·7, 1·9), *P* = 0·321). On the contrary, parents significantly increased their adherence to the Mediterranean diet between baseline and at the end of the 3-month intervention period (increase, 2·1 (0·7, 3·5), *P* = 0·005) and between baseline and at the end of total 6-month maintenance period (increase, 2·9 (1·7, 4·2), *P* < 0·001).

Regarding dietary habits, children significantly increased the consumption of vegetables (*P* = 0·001), legumes (*P* < 0·001) and water (*P* = 0·031) and significantly decreased the consumption of convenience foods (*P* = 0·007) and snacks (*P* = 0·042) over the total 9-month study period (Table 5). In addition, a trend towards significance was observed for an increase in the consumption of fruits (*P* = 0·065) and fish (*P* = 0·061) and a decrease in the consumption of fats other than olive oil (*P* = 0·089). No significant differences were observed in the change of consumption of other food groups (dairy products, nuts, cereals and potatoes, meat and processed meat, eggs, sweets, olive oil and sweet and/or carbohydrate beverages).

**Table 5:** Changes in children’s dietary habits between baseline and the total 9-month follow-up (*n* 13)

	Baseline		Change		*P* value
	Mean	sd	Mean	95 % CI	
Energy (kcal/day)	2211	762·7	29·3	–383·7, 442·3	0·880
Carbohydrates (g/day)	245·6	104·9	8·5	–43·4, 60·4	0·727
Protein (g/day)	91·2	29·7	5·7	–11·1, 22·4	0·473
Fat (g/day)	96·0	31·6	–3·1	–21·3, 15·2	0·721
SFA	27·5	10·7	–0·02	–6·1, 6·1	0·995
MUFA	38·0	11·2	–1·8	–9·2, 5·6	0·611
PUFA	12·7	4·9	–1·8	–4·6, 1·0	0·190
Dairy products (g/day)	459·5	246·9	9·6	–80·4, 99·6	0·820
Fruits (g/day)	165·8	167·4	137·8	–10·2, 285·8	0·065
Vegetables (g/day)	85·7	57·5	234·8	148·3, 321·3	0·001*
Legumes (g/day)	151·9	94·2	74·6	42·0, 107·1	<0·001
Nuts (g/day)	5·6	9·1	2·5	–4·0, 9·0	0·427
Cereals and potato (g/day)	195·8	91·4	–3·3	–53·6, 47·0	0·889
Meat and processed meat (g/week)	1092	476·5	–100·0	–379·8, 179·8	0·451
Fish (g/week)	176·5	96·3	63·5	–3·5, 130·5	0·061
Eggs (g/week)	152·4	57·2	–2·0	–31·5, 27·4	0·884
Sweets (g/day)	63·7	40·1	–1·4	–25·0, 22·1	0·898
Convenience foods (g/week)	318·6	80·1	–131·8	–220·4, –43·2	0·007
Snacks (g/week)	138·1	113·4	–56·2	–110·1, –2·3	0·042
Olive oil (g/day)	19·6	8·5	–0·3	–6·0, 5·4	0·795*
Other fats than olive oil (g/day)	2·6	3·1	–1·2	–2·5, 0·2	0·089
Sweet and/or carbohydrate beverages (ml/week)	36·4	50·0	–1·6	–22·8, 19·5	0·868
Water (ml/day)	947·2	406·2	260·5	27·5, 493·5	0·031

* Wilcoxon matched-pairs signed-rank test.

**Fig. 4:**
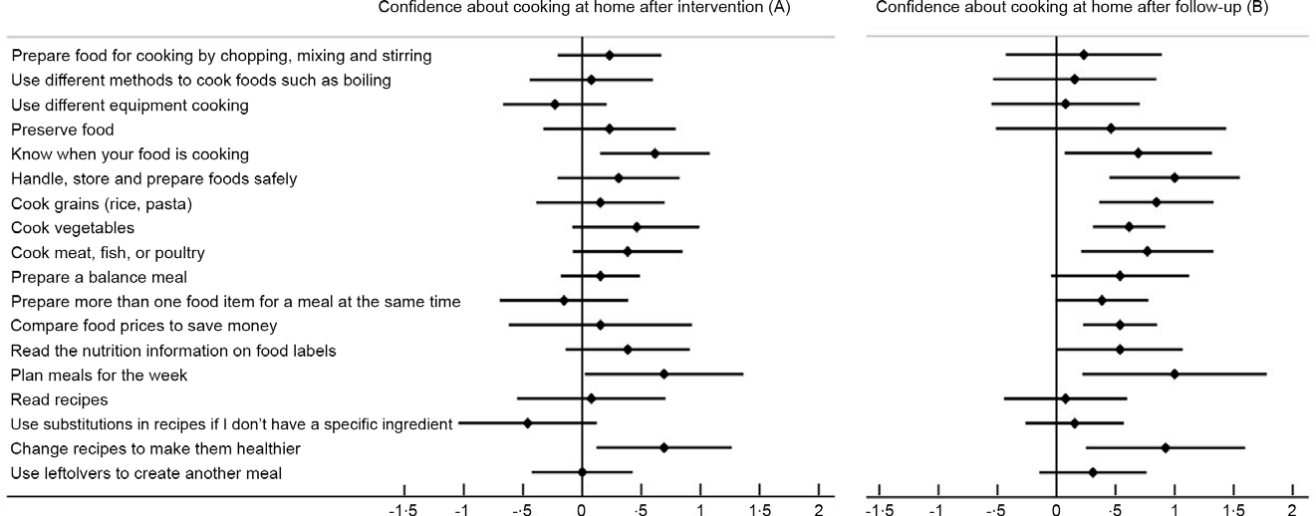
Changes between baseline and (a) after the intervention (3 months) or (b) the total follow-up period (9 months) in parents’ confidence in cooking at home (*n* 13)

Changes in parent’s culinary habits after the 6-month maintenance period are shown in Table 6. In terms of cooking techniques, parents significantly reduced the use of frying (*P* = 0·036) and stewing (*P* = 0·047). In terms of food groups, parents decreased the cooking of white meat (*P* = 0·018), red meat (*P* = 0·002), vegetables (*P* = 0·045) and potatoes and tubers (*P* = 0·024). Parental confidence and attitudes towards cooking at home are shown in Fig. 4 and in online supplementary material, Supplementary Table 1, respectively. After the intervention period, parents significantly increased their confidence in the items ‘knowing when your food is cooked’ (*P* = 0·014), ‘planning meals for the week’ (*P* = 0·046) and ‘changing recipes to make them healthier’ (*P* = 0·015) (Fig. 4(a)). In fact, parents reported significantly higher global confidence scores after the follow-up period (*P* < 0·001) (Fig. 4(b)). In addition to ‘handling, storing and preparing food safely’ (*P* = 0·002), ‘cooking grains’ (*P* = 0·002), ‘cooking vegetables’ (*P* =< 0·001), ‘cooking meat, fish or poultry’ (*P* = 0·011), ‘comparing prices’ (*P* = 0·003) and ‘reading food labels’ (*P* = 0·026) significant increases in confidence were observed in those aspects that improved in the first period.

**Table 6:** Changes in parents’ culinary habits between baseline and the total 9-month follow-up (*n* 13)

	Baseline		After follow-up		*P* value
	*n*	%	*n*	%	
Weekly meal planning					
Planification	8	61·5	11	84·6	0·25
No planification	5	38·5	2	15·4	
In charge of weekly grocery shopping					
No	1	7·7	1	7·7	1·00
Yes	12	92·3	12	9·3	
Cook					
No	1	7·7	2	15·4	1·00
Yes	12	92·3	11	84·6	
Cook days per week					
Not dairy	6	50·0	6	54·5	1·00
Dairy	6	50·0	5	45·5	
Cook hours per week					
≤7 h	9	75·0	11	100·0	0·25
>7 h	3	25·0	0	0·0	

	Baseline		Change		*P* value
	Mean	sd	Mean	95 % CI	
*Use of culinary techniques (times per week)*					
Baking/Roasting	2·9	2·0	–0·9	–2·3, 0·6	0·2181
Grilling (barbecue)	0·3	0·8	–0·1	–0·4, 0·1	0·224*
Grilling (griddle)	3·5	2·6	–0·6	–2·3, 1·1	0·456
Frying	2·5	1·5	–0·8	–1·6, –0·1	0·036
Battered/Breaded and fried	1·2	1·2	–0·6	–1·4, 0·3	0·163
Boiling	8·7	3·2	–0·3	–4·0, 3·3	0·845
Steaming	0·8	0·9	–0·0	–1·0, 0·9	0·963
Stewing	1·7	1·2	–0·7	–1·3, –0·0	0·047
Microwaving	0·3	1·0	0·2	–0·5, 0·8	0·625
Sautéeing	0·3	0·6	–0·1	–0·4, 0·2	0·381*
Raw (vegetables)	2·7	2·0	–0·9	–2·7, 0·9	0·308
Omelet (eggs)	1·7	0·8	0·0	–0·9, 0·9	0·995
*Cooking food groups (times per week)*					
Egg	3·0	1·1	0·6	–0·8, 2·0	0·363
White meat	3·6	0·9	–1·2	–2·1, –0·2	0·018
Read meat	3·6	1·3	–1·6	–2·5, –0·7	0·002
Fish	2·2	1·0	0·1	–0·7, 1·0	0·775
Vegetables	8·4	3·0	–2·4	–4·7, –0·1	0·045
Potato and other tubers	3·5	1·7	–1·2	–2·2, –0·2	0·024
Legumes	1·6	1·0	0·1	–0·8, 0·9	0·844
Cereals	1·6*	0·9	–0·2	–0·8, 0·3	0·390

* Wilcoxon matched-pairs signed-rank test.

In terms of attitudes, parents increased their overall attitude towards cooking at home after the intervention period (*P* = 0·005), although this change did not remain statistically significant after the follow-up period (*P* = 0·103) (see online supplementary material, Supplementary Table 1). Item by item, parents showed a positive change after the intervention on the items ‘It is not important that I know how to cook’ (*P* = 0·026) and ‘It is easy to prepare meals’ (*P* = 0·013) and after the follow-up period on the item ‘It is important to eat the recommended 3 fruits/day’ (*P* = 0·046).

## Discussion

The aim of the present study was to analyse the feasibility, acceptability and preliminary efficacy of a culinary nutritional intervention for parent–child dyads to promote the consumption of plant-based foods in the context of the Mediterranean diet. Overall, the current feasibility study was successful in terms of the acceptability of the culinary nutritional intervention. Significant improvements were also observed in the consumption of some plant-based foods and increased confidence in cooking at home, both in the parents and in the children, during the 3-month intervention period and the additional 6-month follow-up. However, we also found non-significant changes in cooking frequency and use of culinary techniques, suggesting that changes to the intervention are needed before designing a full-scale randomised controlled trial.

In our study, three parent–child dyads dropped out during the follow-up period. This is consistent with previous intervention studies where the dropout rate was around 20–30 %^(48)^. The reason for dropping out before the start of the intervention or before the end of the clinical visits was lack of time due to other children’s activities or the parents’ work. All the parent–child dyads attended all the workshops, except for one family who was unable to attend one of the online workshops. The high rate of workshop attendance could be partly explained by the fact that the face-to-face workshops were individualised for each family and the research group tried to adapt to the family’s schedule. However, it should be noted that workshops 3 and 4 (online workshops) were conducted in groups of five parent–child dyads, and the quality and satisfaction were rated lower by parents and children. These findings support the idea that in future studies with large samples, where individual family workshops are not possible due to lack of time and funding, face-to-face workshops should be limited to a reduced number of parent–child dyads to allow for personalised intervention. In addition, the organisation of the workshops could be improved to fit around family’s schedules.

In terms of quality, time, content and development, the culinary nutritional intervention was generally acceptable to the dyads, according to the *ad hoc* satisfaction questionnaire completed after each workshop. However, parents and children found the online workshop three less interesting, and they emphasised that the combination of recipes and activities was more complicated than in other workshops. In this sense, the parent–child dyads reported that this online workshop was difficult to interact with the chef and the dietitian-nutritionist. Although assessing how to deliver the workshops with only two sessions may be limited, according to our results this problem highlights two important methodological considerations. First, workshops should preferably be face-to-face rather than online. Second, in the case of online workshops, the number of parent–child dyads per group should be limited to three or four families per workshop. However, other studies have found that online cooking workshops are well accepted by children^(49)^ and adults^(50)^. Future research is therefore needed to clarify whether, when and how face-to-face culinary workshops compare favourably with online workshops for child–parent dyads.

Interestingly, both children and parents reported benefits for themselves and their own families in terms of nutrition and cooking aspects. In this sense, children reported an improvement in their taste for plant-based foods. Involving children in home cooking has been suggested as a strategy to improve vegetable liking^(20,51)^. For example, the study by Allirot and cols.^(51)^ found that involving children in cooking activities could increase their willingness to try new foods, including vegetables. In addition, most adult participants agreed that they increased their consumption of vegetables and whole grains and decreased their consumption of convenience foods. These findings are consistent with previous family intervention studies, which found that cooking interventions were associated with improved dietary habits, including increased consumption of plant-based foods and decreased consumption of processed foods^(22,23)^.

Our study also measured changes in the dietary and culinary habits in children and their parents. Children did not significantly change their adherence to the Mediterranean diet, but they increased their consumption of vegetables and legumes. This result is consistent with previous studies in families analysing the association between home cooking and changes in dietary habits^(22,23)^. Interestingly, in our study, the children reported a reduction in their consumption of convenience foods. This reduction may be due to the increased confidence and attitude towards home cooking reported by parents after the intervention. Indeed, a previous study found a negative association between parents’ cooking skills and their children’s consumption of ultra-processed foods^(52)^. In contrast to the results for children, parents’ adherence to the Mediterranean diet increased after the intervention. In the same way, Razavi and cols.^(22)^ showed a higher adherence to the Mediterranean diet in families after a 12-h nutrition and cooking course^(53)^. As a result, encouraging parents and children to cook at home can be a good family-focused public health approach to promoting long-term healthy eating and cooking habits.

Limited cooking attitudes and cooking confidence have been proposed as barriers to home cooking^(6)^. In this regard, parents reported higher cooking attitudes and cooking confidence after the intervention and the follow-up period, respectively. These findings are consistent with previous evaluations of family cooking programmes^(20,24,25)^. To our knowledge, the current study is the first to assess changes in the frequency of use of an extensive list of culinary techniques and the frequency of cooking by food group. Parents reduced frying, probably one of the most commonly used culinary techniques among families because of its palatability and short cooking time, and they reduced the frequency of cooking white and red meats and potatoes and other tubers. However, contrary to our expectations, we found that parents reduced the number of times per week they cooked vegetables. A previous family cooking intervention to increase vegetable consumption has found increased confidence in vegetable preparation when comparing pre- and post-intervention data^(20)^. However, this study did not measure the frequency with which families prepared vegetables. Therefore, more research is needed to confirm that cooking interventions increase the frequency with which participants cook vegetables and other food groups.

There are several limitations to this feasibility study. First, since there was no control group, we could not compare the effectiveness of the culinary nutritional intervention with a traditional nutritional intervention. Second, although preliminary analysis showed significant changes in dietary and culinary habits, the trend of results in such a small sample size study should be interpreted with caution. Third, due to resource constraints, we did not measure children’s cooking confidence and skills, and we did not collect an FFQ from parents. However, we measured the adherence to the Mediterranean diet. Fourth, we used self-reported data rather than the use of biomarkers. Nevertheless the questionnaires have been previously validated^(39,46,54)^. Fifth, the recruitment of families only among the employees of the University of Navarra limits the generalisability of the results to other populations. However, we observed similar proportions of children with low, moderate or high adherence to the Mediterranean diet at baseline to those reported previously in Spanish children^(55,56)^. Sixth, due to the large disparity in the number of girls and boys recruited (12 *v*. 3, respectively), it was not possible to present key findings on the feasibility, acceptability and effectiveness of the intervention by sex. Stratifying the randomisation by sex of the children will be important in future studies. Seventh, the n-CHEF intervention focuses on changing dietary behaviour at the individual and family level. However, we recognise that changing dietary habits is a multi-level approach and the impact of the intervention may be limited. Despite these limitations, the study has several strengths. First, the provision of multiple options to follow-up the workshops helped to overcome barriers related to unreliable work schedules and children’s schedules and was crucial to the high retention rate during the follow-up. Second, the culinary nutritional intervention was based on the ten experiential drivers of behaviour change in culinary nutritional interventions identified by Fredericks and cols.^(35)^ Finally, the study included a post-intervention follow-up period, which allowed us to measure changes in dietary and culinary habits after a total of 9 months.

In conclusion, the n-CHEF feasibility study provides insight into feasibility and patient acceptability of the culinary nutritional intervention. In addition, the hands-on family cooking and nutrition workshops appear to have improved the dietary and culinary habits of the parent–child dyads. These findings will support future randomised control trials to test whether a family culinary and nutritional intervention can improve dietary habits and reduce the burden of childhood obesity and other chronic diseases. In this sense, we have conducted the n-CHEFS a randomised multicentre pilot study to analyse the effect on dietary habits of a culinary nutritional intervention for parent–child dyads compared with a culinary nutritional intervention only for parents or a nutritional intervention for parent–child dyads (ClinicalTrials.gov NCT05280652).

## Supporting information

Goni et al. supplementary material Goni et al. supplementary material

## Supplementary material

For supplementary material accompanying this paper visit https://doi.org/10.1017/S1368980024001538.
