# Supplementary material for: Nutritional and Culinary Habits to Empower Families (n-CHEF): a feasibility study to increase consumption and home cooking of plant-based foods
Source: Public Health Nutr. 2024 Oct 1;27(1):e190. doi: 10.1017/S1368980024001538 (PMC11504476; doi:10.1017/S1368980024001538)
Supplement: Goni et al. supplementary material [file S1368980024001538sup001.docx]

**SUPPLEMENTARY MATERIAL**

**Supplementary Table 1.** Changes in parents’ attitudes about cooking at home between baseline and after the intervention or the follow-up period (n=13)

|  | **Intervention period** | | | **Follow-up period** | | |
| --- | --- | --- | --- | --- | --- | --- |
|  | Baseline  Mean (SD) | Change  Mean (95%CI) | *P*  value | Baseline  Mean (SD) | Change  Mean (95%CI) | *P* value |
| Global attitude about cooking at home | 74.5 (7.2) | 4.0 (15;6.5) | 0.005 | 74.4 (7.1) | 3.1 (-0.7;7.1) | 0.103 |
| **Attitude in the use of specific cooking techniques and skills** | | | |  |  |  |
| I do NOT like to cook because it takes too much time | 4.5 (0.9) | -0.1 (-0.7;0.4) | 0.584 | 4.5 (0.9) | 0.0 (-0.2;0.2) | 1.000 |
| Preparing meals at home would NOT improve the health of my diet | 4.4 (1.1) | 0.5 (-0.2;1.1) | 0.084* | 4.4 (1.1) | 0.5 (-0.3;1.2) | 0.169* |
| Cooking meals is a good use of my time | 3.8 (1.3) | 0.4 (-0.1;0.8) | 0.096 | 3.7 (1.2) | 0.4 (-0.1;0.8) | 0.084* |
| I enjoy cooking | 4.3 (1.2) | 0.0 (-0.3;0.3) | 1.000 | 4.3 (1.2) | 0.0 (-0.3;0.3) | 1.000 |
| It is important to know how to prepare food | 4.6 (0.5) | 0.1 (-0.2;0.5) | 0.337 | 4.6 (0.5) | 0.0 (-0.2;0.2) | 1.000 |
| Cooking is fun | 4.1 (0.9) | 0.1 (-0.3;0.6) | 0.501 | 4.0 (0.9) | 0.3 (-0.1;0.8) | 0.165 |
| I do NOT like to prepare meals at home because it costs too much money | 4.6 (0.8) | 0.2 (-0.2;0.7) | 0.298* | 4.5 (0.8) | -0.1 (-0.9;0.8) | 1.000* |
| It is NOT important that I know how to cook. | 3.9 (1.2) | 0.8 (-0.0;1.5) | 0.026* | 4.1 (1.3) | 0.7 (-0.1;1.5) | 0.090* |
| Cooking is interesting | 4.4 (0.6) | 0.1 (-0.2;0.5) | 0.337 | 4.4 (0.6) | -0.1 (-0.5;0.4) | 0.721 |
| Meals made at home are affordable | 4.5 (0.7) | 0.2 (-0.2;0.7) | 0.298* | 4.5 (0.7) | -0.1 (-0.5;0.3) | 0.673 |
| It is important to eat the recommended 3 portions of fruit each day | 4.2 (0.8) | 0.1 (-0.2;0.5) | 0.337 | 4.2 (0.8) | 0.4 (-0.0;0.8) | 0.046* |
| It is important to eat the recommended 2 portions of vegetables each day | 4.2 (0.7) | 0.2 (-0.1;0.6) | 0.190 | 4.3 (0.7) | 0.0 (-0.8;0.8) | 0.389* |
| It is easy to prepare meals | 3.5 (0.9) | 0.6 (0.1;1.1) | 0.013 | 3.5 (0.9) | 0.4 (-0.1;0.9) | 0.137 |
| Cooking is frustrating | 4.4 (0.5) | -0.1 (-0.5;0.3) | 0.673 | 4.4 (0.5) | -0.1 (-0.7;0.5) | 0.794 |
| I like trying new recipes | 4.1 (0.9) | 0.2 (-0.1;0.6) | 0.190 | 4.1 (0.9) | 0.2 (-0.2;0.7) | 0.273 |
| It is too much work to cook | 2.5 (1.0) | 0.3 (-0.3;0.9) | 0.303 | 2.4 (0.9) | 0.4 (-0.2;1.0) | 0.209 |
| Making meals at home helps me to eat  more healthfully | 4.7 (0.5) | 0.0 (-0.3;0.3) | 1.000 | 4.7 (0.5) | -0.1 (-0.4;0.2) | 0.564* |
| I find cooking tiring | 3.8 (0.9) | 0.1 (-0.5;0.8) | 0.613 | 3.7 (0.8) | 0.3 (-0.3;0.9) | 0.264 |

95%CI, 95% Confidence interval; SD, Standard deviation

*Wilcoxon matched-pairs signed-rank test
